# Programmable scanning diffuse speckle contrast imaging of cerebral blood flow

**DOI:** 10.1117/1.NPh.12.1.015006

**Published:** 2025-01-27

**Authors:** Faezeh Akbari, Xuhui Liu, Fatemeh Hamedi, Mehrana Mohtasebi, Li Chen, Lei Chen, Guoqiang Yu

**Affiliations:** a University of Kentucky, Department of Biomedical Engineering, Lexington, Kentucky, United States; b University of Kentucky, Biostatistics and Bioinformatics Shared Resource Facility, Markey Cancer Center, Lexington, Kentucky, United States; c University of Kentucky, Spinal Cord and Brain Injury Research Center, Department of Physiology, Lexington, Kentucky, United States

**Keywords:** diffuse optics, speckle contrast imaging, digital micromirror device, line-shaped scanning, cerebral blood flow

## Abstract

**Significance:**

Cerebral blood flow (CBF) imaging is crucial for diagnosing cerebrovascular diseases. However, existing large neuroimaging techniques with high cost, low sampling rate, and poor mobility make them unsuitable for continuous and longitudinal CBF monitoring at the bedside.

**Aim:**

We aimed to develop a low-cost, portable, programmable scanning diffuse speckle contrast imaging (PS-DSCI) technology for fast, high-density, and depth-sensitive imaging of CBF in rodents.

**Approach:**

The PS-DSCI employed a programmable digital micromirror device (DMD) for remote line-shaped laser (785 nm) scanning on tissue surface and synchronized a 2D camera for capturing boundary diffuse laser speckle contrasts. New algorithms were developed to address deformations of line-shaped scanning, thus minimizing CBF reconstruction artifacts. The PS-DSCI was examined in head-simulating phantoms and adult mice.

**Results:**

The PS-DSCI enables resolving intralipid particle flow contrasts at different tissue depths. *In vivo* experiments in adult mice demonstrated the capability of PS-DSCI to image global/regional CBF variations induced by 8% ${\mathrm{CO}}_{2}$ inhalation and transient carotid artery ligations.

**Conclusions:**

Compared with conventional point scanning, line scanning in PS-DSCI significantly increases spatiotemporal resolution. The high sampling rate of PS-DSCI is crucial for capturing rapid CBF changes while high spatial resolution is important for visualizing brain vasculature.

## 1 Introduction

Cerebral blood flow (CBF) plays a critical role in sustaining brain health and function. CBF serves as the conduit for delivering essential oxygen and nutrients to the brain while facilitating the removal of waste products.^1^^–^^3^ The vitality and functionality of the brain are intricately linked to the efficiency of blood circulation and hemodynamic processes. Continuous and longitudinal monitoring of CBF is crucial for understanding the pathophysiology and developing treatment strategies for many cerebral/neurovascular diseases including stroke,^4^^–^^6^ intraventricular hemorrhage,^7^^,^^8^ and traumatic brain injury.^9^^,^^10^

Various modalities exist for cerebral hemodynamic imaging. Magnetic resonance imaging (MRI)^11^^–^^13^ and positron emission tomography (PET)^14^^,^^15^ offer the capability of whole-head imaging of brain hemodynamics and metabolism. However, the high cost, low sampling rate, and poor mobility make them unsuitable for continuous and longitudinal cerebral monitoring at the bedside. Optical imaging modalities present a compelling alternative due to their safety, portability, mobility, affordability, and high temporal resolution. Laser speckle contrast imaging (LSCI) is a noncontact imaging tool with high spatiotemporal resolution but limited penetration depth (less than 1 mm) because of using wide-field illumination.^16^^,^^17^ Laser Doppler flowmetry,^18^^,^^19^ diffuse correlation spectroscopy,^20^^–^^22^ and diffuse speckle contrast flowmetry^23^^–^^25^ utilize limited discrete source-detector (S-D) pairs in contact with the head for point-of-care measurements of CBF. The sparse arrangement of S-D pairs inherently limits spatial resolutions. Increasing the number of sources and detectors escalates instrumentation costs and slows downsampling rates. Moreover, the contact measurement may be impractical for intraoperative monitoring of wounded/injured tissues.

To overcome the limitations of existing technologies, researchers have prompted the development of noncontact optical imaging devices with scanning illumination and high-resolution 2D cameras for high-density imaging of blood flow distributions.^26^^–^^28^ Speckle contrast diffuse correlation tomography (scDCT)^29^ is one of these modalities that we have previously developed for high-density imaging of CBF distributions.^29^^–^^37^ In scDCT, a galvo mirror remotely scans a near-infrared coherent point source to multiple positions on a selected region of interest (ROI).^38^ A high-resolution scientific complementary metal oxide semiconductor (sCMOS) camera, serving as a 2D detector array, captures fluctuations of spatial diffuse laser speckles resulting from motions of red blood cells in the measured tissue volume (i.e., tissue blood flow). Based on photon diffusion theories, the maximum tissue penetration depth is approximately one half of the S-D separation used.^39^^–^^41^ Therefore, tissue blood flow distributions at different depths are reconstructed by quantifying spatial laser speckle contrasts in selected detector areas, defined at specific S-D separations. Continuous and longitudinal imaging of CBF distributions has been successfully demonstrated using the scDCT in rodents, piglets, and human subjects.^29^^–^^36^ Although effective, scDCT requires scanning of a point source to numerous positions for high-density sampling, which is time-consuming.^42^ This long scanning time limits the scDCT for applications wherein a high temporal resolution is needed, such as imaging brain functional connectivity (FC).^43^^–^^46^

To improve scanning efficiency and spatiotemporal resolution, we developed a programmable scanning diffuse speckle contrast imaging (PS-DSCI) technology for rapid and high-density imaging of CBF distributions at different tissue depths. The innovative PS-DSCI employed a programmable digital micromirror device (DMD) for remote line-shaped laser scanning on the tissue surface, synchronized with a high-resolution 2D camera to capture boundary diffuse laser speckle contrasts. Although line scanning can be achieved through various methods, including line lasers^47^ and a combination of a cylindrical lens with a galvo mirror,^48^ the programmable DMD maximizes operational flexibility, allowing for the adjustment to scanning patterns according to specific applications. New algorithms were developed to analyze the collected PS-DSCI data for 2D mapping of blood flow distributions at different depths. The PS-DSCI system was evaluated using head-simulating phantoms with known optical and flow properties. Finally, *in vivo* experiments in adult mice demonstrated the capability of the PS-DSCI system to image global and regional CBF variations induced by 8% ${\mathrm{CO}}_{2}$ inhalation and transient carotid artery ligations.

## 2 Methods

### 2.1 PS-DSCI System

#### 2.1.1 PS-DSCI principle and prototype

**Fig. 1:**
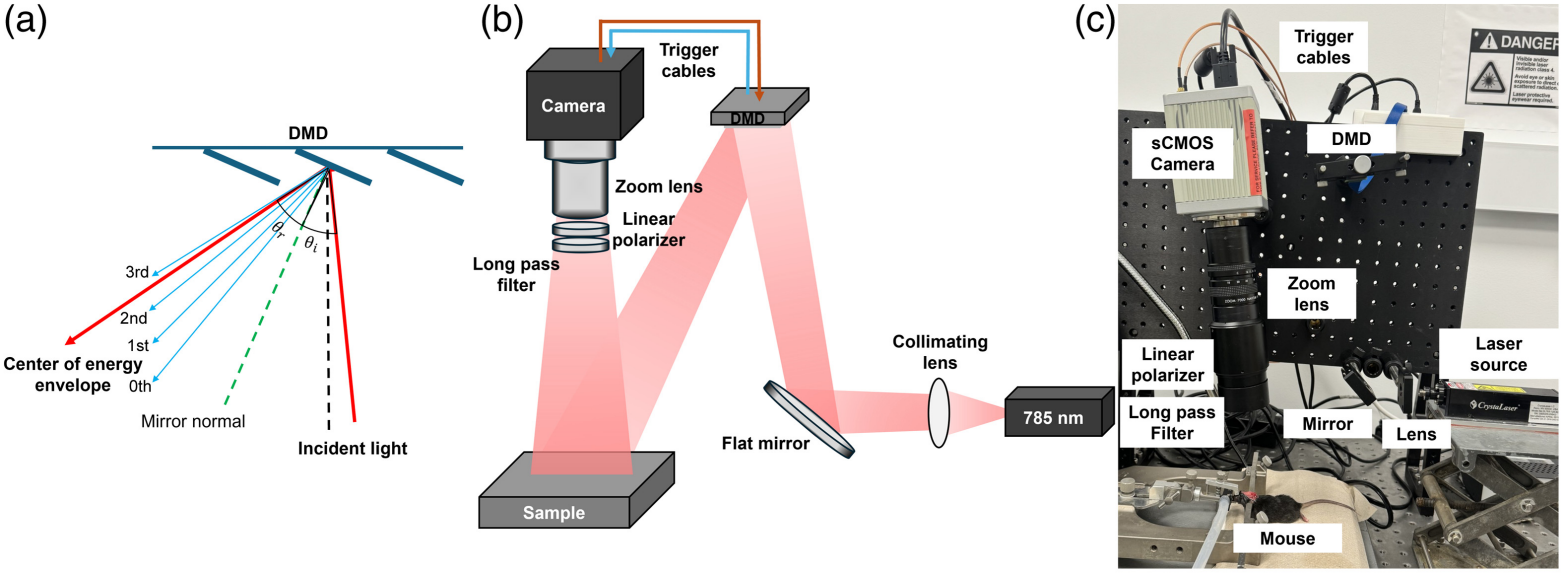
PS-DSCI system. (a) The distribution of diffraction orders and energy envelope reflected by the DMD, which are dependent on the angle of incidence (θi) and angle of reflection (θr). θr represents the center of energy envelope distribution. (b) Schematic of the PS-DSCI prototype. (c) A photo of the PS-DSCI prototype.

In the PS-DSCI prototype (Fig. 1), an open-space linearly polarized coherent laser (785 nm, 120 mW, DL785-120-S, CrystaLaser)^38^ illuminates a semi-collimated light via a biconvex lens (LB1092-B, Thorlabs, Newton, United States) and a flat mirror (UM10-AG, Thorlabs) onto the micromirror array of a programmable DMD (DLP4500, Texas Instruments, Dallas, United States) to achieve rapid line scanning. Although using a projection lens after the DMD can improve spatial resolution and enhance light power efficiency, it limits the working distance and requires precise alignment for each measurement to superimpose different diffraction orders on the target tissue. By contrast, semi-collimated illumination offers a larger depth of focus, enabling line-shaped laser scanning over the selected ROI at varied working distances. In addition, collimated light ensures that different diffraction orders are well separated by maximizing their angular separations, thus preventing overlap in the scanning area.^49^ The incident angle was optimized to align the brightest diffraction order with the center of the energy envelope to maximize the single-order diffraction efficiency [Fig. 1(a)].

A sCMOS camera (C11440-42U40, Hamamatsu, Japan), connected to an adjustable zoom lens (Zoom 7000, Navitar), was synchronized with the DMD to sequentially capture raw intensity images of the line scanning illumination on the ROI. A linear polarizer (LPNIRE200-B, Thorlabs) and a long-pass filter (84–761, Edmund Optics, Barrington, United States) were installed in front of the zoom lens to reduce specular reflection and ambient light, respectively. All components were installed on a vertically mounted optical breadboard to facilitate animal experiments [Figs. 1(b) and 1(c)]. This compact setup allows for assembling the PS-DSCI system on a portable, movable cart to facilitate bedside measurements.

#### 2.1.2 Transition from point scanning to line scanning

**Fig. 2:**
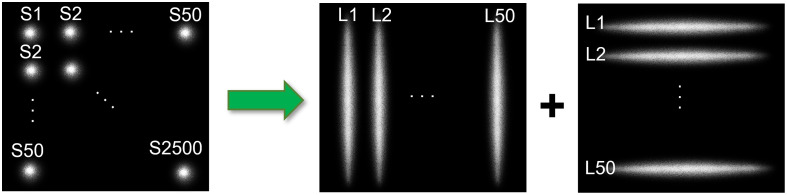
Point scanning (scDCT) versus line scanning (PS-DSCI). In contrast to 2500 scanning points (S1 to S2500) by scDCT, PS-DSCI scans the same ROI with 100 scanning lines (L1 to L50, along vertical and horizontal directions, respectively).

Compared with point source scanning in scDCT, line scanning in PS-DSCI is more efficient in terms of sampling rate and number of images acquired, processed, and stored (Fig. 2). Table 1 summarizes the remarkable improvements from the point source scanning (scDCT) to line scanning approach (PS-DSCI) over the same ROI. Using the same camera with a frame rate of 24 fps (C11440-42U40, Hamamatsu) for both setups, the sampling rate increases from 0.0096 Hz (total 50×50 scanning points in scDCT) to 0.24 Hz (total 50 + 50 scanning lines in PS-DSCI), representing a ∼25-fold improvement achieved by the PS-DSCI with total 100 scanning lines, used in the present study. Line scanning significantly reduces the number of raw intensity images acquired for blood flow reconstruction, leading to a 23-fold reduction in computation time and a 25-fold reduction in data storage. The larger the number of scanning points/lines, the higher the working efficiency.

**Table 1:** Comparisons between the point scanning and line scanning.

Methods	Number of images	Sampling rate (Hz)	Computation time (s)	Storage (megabytes)
Point scanning	2500 (50 × 50 points)	0.0096	∼410	20,000
Line scanning	100 (50 + 50 lines)	0.24	∼17	800

### 2.2 Data Analysis

#### 2.2.1 Automatic extraction of detector area/belt around line-shaped source

**Fig. 3:**
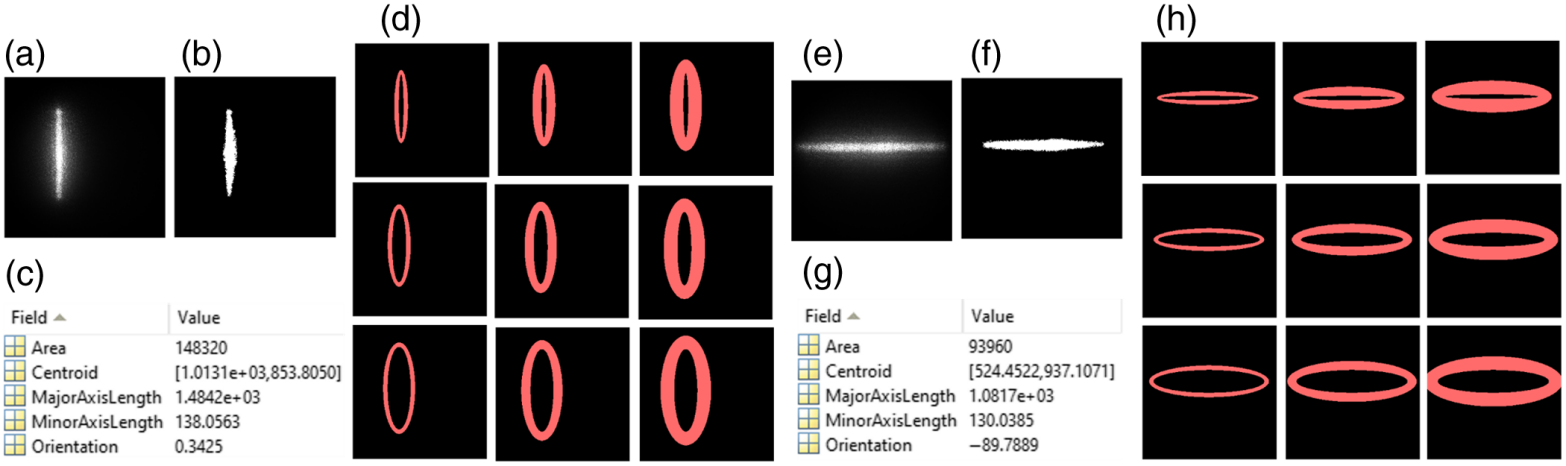
Extraction of detector area/belt around the line-shaped source. (a) and (e) Raw intensity images of vertical and horizontal oval-shaped sources, respectively. (b) and (f) Binary masks of vertical and horizontal oval-shaped sources, respectively. (c) and (g) An example of vertical and horizontal oval-shaped source properties extracted using the “regionprops” function in MATLAB’s Image Processing Toolbox. (d) and (h) The elliptical-shaped detector belts with varied S-D separations of 1 to 3 mm and varied detector-belt thicknesses of 1 to 3 mm.

Our innovative approach for depth-sensitive blood flow reconstruction involves defining detector areas located at certain distances from the source center, with the goal of selectively capturing diffused photons originating from certain depths. The synchronization between the DMD and sCMOS camera ensures the capture of one intensity image at each source position. However, the line shapes of scanning sources typically transform into oval shapes due to the Gaussian distribution of the light source and the curvature of the target tissue surface [Figs. 3(a)–3(c) and 3(e)–3(g)]. We innovatively developed a new algorithm in MATLAB to extract the source properties at each scanning position and then used these properties to generate a detector area/belt with a predefined S-D separation [Figs. 3(d) and 3(h)].

Specifically, the captured intensity images were binarized and labeled, and morphological analyses were conducted using the “regionprops” function in MATLAB to identify the properties of oval-shaped sources. The modifiable elliptical-shaped detector belts matching oval-shaped sources were then defined by substituting the extracted properties into the equation of an ellipse [Eq. (1)]:

$$\frac{(x-h{)}^{2}}{a^{2}}+\frac{(y-k{)}^{2}}{b^{2}}=1,$$ (1)

where x and y are rows and columns of the intensity image, respectively, h and k are the center of the oval-shaped source, and a and b are major axis length or minor axis length, depending on the orientation of the source. These processes adaptively configured the detector belt, centered around the oval-shaped source with the same S-D separation [Figs. 3(d) and 3(h)]. The minor and major axis lengths (a and b) can be defined to adjust the S-D separation and detector-belt thickness.

#### 2.2.2 2D Mapping of Blood Flow Index (BFI)

The diffuse laser speckle contrast $\left(K_{s}\right)$ is calculated within the defined detector belt using Eq. (2)^50^^,^^51^

$$K_{s}=\frac{\sigma_{s}}{\left\langle I\right\rangle }=\frac{\sqrt{{\left\langle I\right\rangle }^{2}-{\left\langle I\right\rangle }^{2}}}{\left\langle I\right\rangle },$$ (2)

where $K_{s}$ is the ratio of standard deviation ($\sigma_{s}$) over mean intensity (⟨I⟩) in an N×N (e.g., 3×3, 5×5, or 7×7) pixel window. A 2D matrix of $K_{s}$ values are then generated by aggregating $K_{s}$ values of all line scanning images while normalizing the overlapped detector belts. The BFI is approximated as $\mathrm{BFI}=\frac{1}{{K_{s}}^{2}}$.^52^^,^^53^ Note that the detector belts outside the ROI corresponding to the edge illumination lines are cropped and discarded from the reconstructed BFI maps to eliminate edge artifacts (Fig. 3). Following our previous method, convolution functions and kernel matrixes in MATLAB are used to improve computation efficiency.^42^ Presently, the computation time for processing 100 scanning lines (Table 1) is ∼17  s, which can be further reduced by GPU computing and leveraging MATLAB’s Parallel Computation Toolbox.^54^

### 2.3 Head-Simulating Phantom Experiments

#### 2.3.1 Fabrication of head-simulating phantoms

**Fig. 4:**
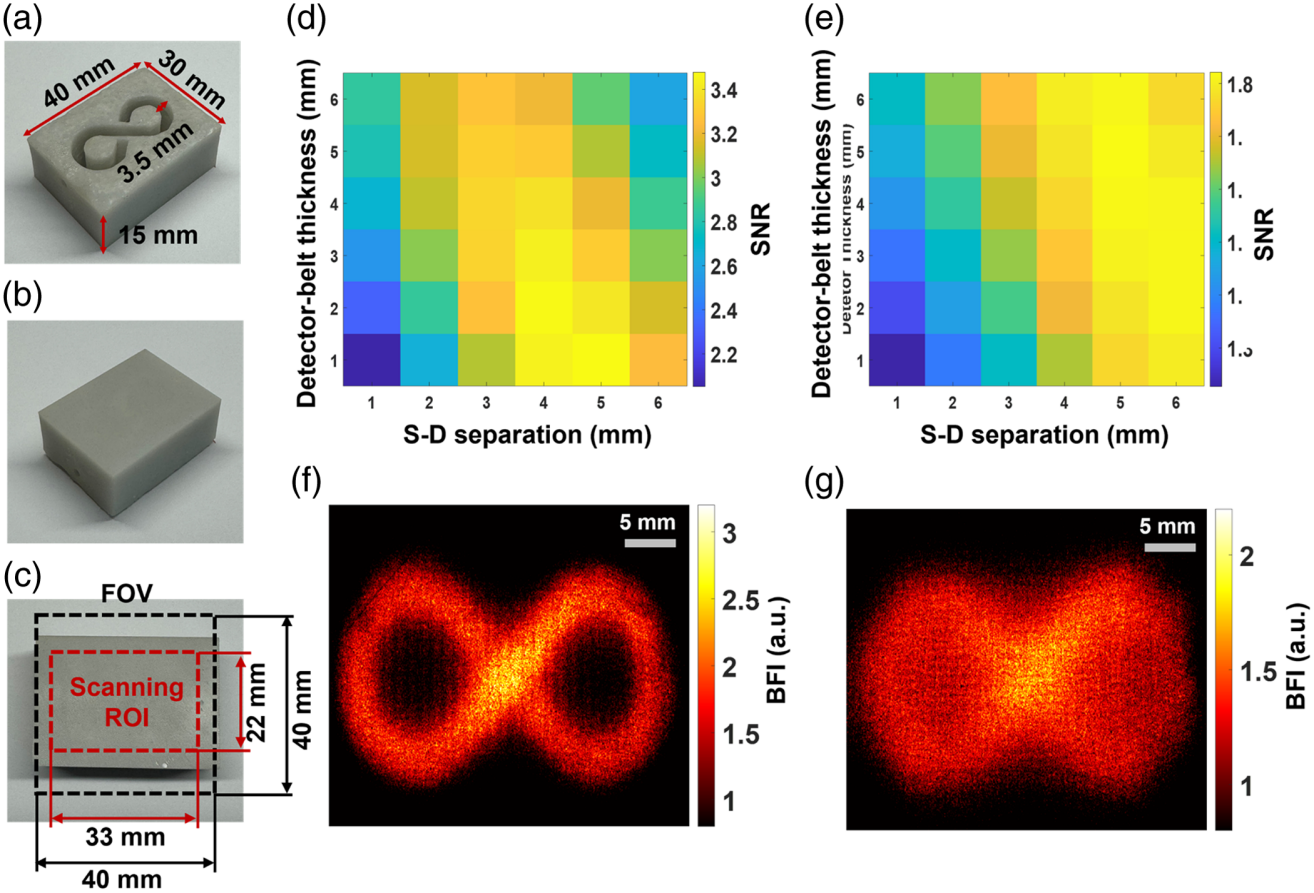
Depth-sensitive 2D mapping of intralipid particle flow in head-simulating phantoms. (a) Bottom view of fabricated solid phantom with the infinity-shaped channel, designed to be filled with the liquid phantom. (b) Top view of the fabricated phantom. (c) The selected camera FOV and scanning ROI on the top of the phantom. (d) The SNR distribution with varied S-D separations (1 to 6 mm) and detector-belt thicknesses (1 to 6 mm) for the phantom with top layer thickness of 1 mm. (e) The SNR distribution with varied S-D separations (1 to 6 mm) and detector-belt thicknesses (1 to 6 mm) for the phantom with top layer thickness of 2 mm. (f) The 2D flow map of head-simulating phantom with the top layer of 1 mm thickness. The S-D separation and detector-belt thickness for flow map reconstruction were 4 and 2 mm, respectively. (g) The 2D flow map of the head-simulating phantom with the top layer of 2 mm thickness. The S-D separation and detector-belt thickness for flow map reconstruction were 5 and 4 mm, respectively.

Standardized tissue-simulating phantoms with known optical properties and geometries are widely accepted for evaluating optical imaging technologies. Following our established methods,^42^^,^^55^ two solid phantoms with infinity-shaped channels were printed (3D printer SL1, Prusa) with the top layer thicknesses of 1 and 2 mm, respectively, to mimic the mouse skull with varied thicknesses. Figures 4(a)–4(c) show the fabricated solid phantom (dimensions: $30\times 40\times 15\;mm^{3}$), incorporating an infinity-shaped channel. The infinity-shaped channel was filled with the liquid phantom solution and sealed by a thin layer of plastic and hot glue. The solid phantoms made of titanium dioxide (${\mathrm{TiO}}_{2}$), India ink (Black India, Massachusetts, United States), and clear resin (eSUN Hard Tough) were designed to accommodate the liquid phantom solution composed of Intralipid (Fresenius Kabi, Sweden), Indian ink, and water. The concentration of Indian ink controlled the tissue absorption coefficient ($\mu_{a}$), whereas ${\mathrm{TiO}}_{2}$ and intralipid concentrations regulated the reduced scattering coefficient ($\mu_{s}^{\prime }$). The Brownian motion of intralipid particles within the infinity-shaped channel simulated particle flow to mimic the motion of red blood cells in vessels (i.e., blood flow). The optical properties of both the solid and liquid phantoms were set at $\mu_{a}=0.03\;{\mathrm{cm}}^{-1}$ and $\mu_{s}^{\prime }=9\;{\mathrm{cm}}^{-1}$.

#### 2.3.2 Experimental setup and procedures

Each phantom was scanned with a total of 100 lines: 50 vertical and 50 horizontal lines on the selected ROI of $33\times 22\;{\mathrm{mm}}^{2}$. The camera collected 100 images on the selected field of view (FOV) of $40\times 40\;{\mathrm{mm}}^{2}$ at a working distance of 150 mm with an exposure time of 10 ms and frame rate of 24 fps (equivalent sampling rate of 0.24 Hz). With an f/# of 8 and a magnification (M) of 0.32, the speckle size ($\rho_{\text{speckle}}$) is 20.2  μm. This speckle size is sufficiently larger than the camera pixel size (6.5  μm) to satisfy the Nyquist condition at 785 nm based on Eq. (3).^16^ Here, M represents the magnification, f/# denotes the f-number of the zoom lens, and λ is the laser wavelength

$$\rho_{\text{speckle}}=2.44(M+1)*\lambda *f/\#.$$ (3)

#### 2.3.3 Signal-to-noise ratio (SNR) analysis

We experimentally evaluated the reconstructed flow imaging quality of head-simulating phantoms by analyzing the raw images with different pixel windows including 3×3, 5×5, 7×7, and 9×9. A window size of 5×5 was finally selected for calculating $K_{s}$ to balance between the spatial resolution and SNR. The detector-belt thickness and S-D separation varied from 1 to 6 mm, respectively, to investigate their impacts on SNRs of flow images. To quantify the SNR, a binary mask was created, representing the known infinity-shaped flow channel. The SNR was calculated by taking the ratio of averaged flow values over the regions with intralipid particle flow of the liquid phantom (valid flow signal, mask = 1) and without particle flow of the solid phantom outside the infinity-shaped channel (noise, mask = 0).

### 2.4 *In Vivo* Experiments in Adult Mice

#### 2.4.1 Experimental setup and procedures

All experimental procedures involving animals were approved by the University of Kentucky Institutional Animal Care and Use Committee (IACUC). Nine adult male mice aged from 9 to 18 weeks with different experimental procedures were imaged by the PS-DSCI (Table 2). The mouse was subjected to 1% to 2% isoflurane anesthesia, and its hairs on the head and at the cervical surgical site were removed by hair removal cream. The surgical skin was disinfected with betadine followed by 70% ethanol. The scalps of eight mice (mouse #1 to mouse #8) were surgically removed to reduce the partial volume effects of scalps on CBF maps. The head of one mouse (mouse #9) was kept intact (without scalp retraction) to test the capability of PS-DSCI penetrating through the intact head. The experimental setup was the same as that used for phantom experiments (2.3.2), except that the FOV and ROI were set up as $30\times 30\;{\mathrm{mm}}^{2}$ and $27\times 18\;{\mathrm{mm}}^{2}$, respectively. This setup resulted in a magnification of 0.43 and a speckle size of 21.9  μm, satisfying the Nyquist condition [Eq. (3)]. Two-dimensional maps of BFI were reconstructed at different depths by calculating $K_{s}$ on a window size of 5×5. The relative time-course changes in CBF (rCBF) were calculated by normalizing BFI data to their baseline values before pathophysiological manipulations.

**Table 2:** Subject information and experimental protocols.

Subjects	Age (weeks)	Experimental protocols	Scalp retraction
Mouse #1	18	8% ${\mathrm{CO}}_{2}$ inhalation, carotid artery ligation	Yes
Mouse #2	18	8% ${\mathrm{CO}}_{2}$ inhalation, carotid artery ligation	Yes
Mouse #3	18	8% ${\mathrm{CO}}_{2}$ inhalation, carotid artery ligation	Yes
Mouse #4	18	8% ${\mathrm{CO}}_{2}$ inhalation, carotid artery ligation	Yes
Mouse #5	18	8% ${\mathrm{CO}}_{2}$ inhalation, died during carotid artery ligation	Yes
Mouse #6	9	8% ${\mathrm{CO}}_{2}$ inhalation, carotid artery ligation	Yes
Mouse #7	9	8% ${\mathrm{CO}}_{2}$ inhalation, carotid artery ligation	Yes
Mouse #8	9	8% ${\mathrm{CO}}_{2}$ inhalation, carotid artery ligation, 100% ${\mathrm{CO}}_{2}$ inhalation	Yes
Mouse #9	9	8% ${\mathrm{CO}}_{2}$ inhalation, carotid artery ligation	No

#### 2.4.2 Continuous imaging of global rCBF in mice during 8% ${\mathrm{CO}}_{2}$ inhalations

${\mathrm{CO}}_{2}$ is a vasodilator, leading to a global increase in rCBF.^56^ The mouse on a heating blanket was anesthetized (1% to 2% isoflurane) with its head secured on a stereotaxic frame. The PS-DSCI imaging was performed for nine mice (eight mice with intact skulls but retracted scalps, and mouse #9 with an intact scalp and skull) before, during, and after a 5-min exposure to a gas mixture consisting of 8% ${\mathrm{CO}}_{2}$ and 92% $O_{2}$.

#### 2.4.3 Continuous imaging of regional rCBF in mice during sequential transient carotid artery ligations

After recovering from 8% ${\mathrm{CO}}_{2}$ inhalation, the mouse underwent sequential transient carotid arterial ligation surgeries to create sequential decreases in rCBF in the left hemisphere (LH) and right hemisphere (RH). Transient carotid arterial ligation surgery involves creating a midline incision to access both carotid arteries. A 6-0 braided nylon loose knot suture was placed around each carotid artery, allowing transient ligation of specific carotid arteries by applying a mild pull on the corresponding knot.^42^^,^^55^ The PS-DSCI imaging was performed for nine mice (eight mice with intact skulls but retracted scalps, and mouse #9 with an intact scalp and skull) during 5 min of baseline, 3 min of right carotid artery ligation, 1 min of bilateral ligation, 3 min of left carotid artery release, and 5 min of right carotid artery release. At the end of the study, one mouse (mouse #8) was subjected to 100% ${\mathrm{CO}}_{2}$ inhalation to induce a sharp reduction in rCBF.

#### 2.4.4 Statistical analysis

Statistical analysis was performed using a two-tailed Wilcoxon signed-rank test in IBM SPSS Statistics software to compare rCBF variations at different phases of 8% ${\mathrm{CO}}_{2}$ inhalation and transient carotid artery ligations. The mean values of rCBF at different phases were calculated for all subjects and compared with their baseline values. A p-value of <0.05 was considered statistically significant for all statistical analyses.

## 3 Results

### 3.1 PS-DSCI Enabled Depth-sensitive 2D Mapping of Particle Flow in Head Simulating Phantoms

Figure 4 shows the fabricated head-simulating phantoms [Figs. 4(a)–4(c)], SNR analysis results [Figs. 4(d) and 4(e)], and reconstructed 2D flow maps [Figs. 4(f) and 4(g)]. Figures 4(d) and 4(e) show SNR distributions with varied S-D separations and detector-belt thicknesses for the two phantoms with top layer thicknesses of 1 and 2 mm, respectively. For the phantom with a top layer thickness of 1 mm, higher SNR values appeared with the S-D separations of 3 to 5 mm and detector-belt thicknesses of 1 to 3 mm [Fig. 4(d)]. For the phantom with a top layer thickness of 2 mm, higher SNR values shifted to larger S-D separations of 4 to 6 mm and thicker detector-belt thicknesses of 2 to 5 mm [Fig. 4(e)]. Figure 4(f) shows the resulting 2D flow map of the phantom with a top layer thickness of 1 mm, reconstructed at the S-D separation of 4 mm and the detector-belt thickness of 2 mm. Similarly, Fig. 4(g) shows the resulting 2D flow map of the phantom with a top layer thickness of 2 mm, reconstructed at the S-D separation of 5 mm and the detector-belt thickness of 4 mm.

These results demonstrated the depth sensitivity of PS-DSCI measurements because larger S-D separations of 4 to 6 mm correspond to deeper imaging depths of 2 to 3 mm as compared with the smaller S-D separations of 3 to 5 mm at a shallower imaging depth of 1.5 to 2.5 mm. Deeper penetration, achieved by utilizing larger S-D separations, resulted in fewer detected diffused photons. In addition, the increased partial volume effect from the thicker top layer (2 mm) of the phantom further contributed to the reduction in detected diffusive photons, leading to lower SNRs. To conclude, our PS-DSCI enabled resolving the infinity-shaped channel of Intralipid particle flow contrasts at the depths up to ∼3  mm in the two phantoms with top layer thicknesses of 1 and 2 mm. These phantom experiments provide guidance for optimizing S-D separations and detector-belt thicknesses to obtain the best image quality based on target head geometries.

### 3.2 *In Vivo* Test Results

#### 3.2.1 PS-DSCI enabled 2D mapping of BFI at different depths in mice with intact skull

**Fig. 5:**
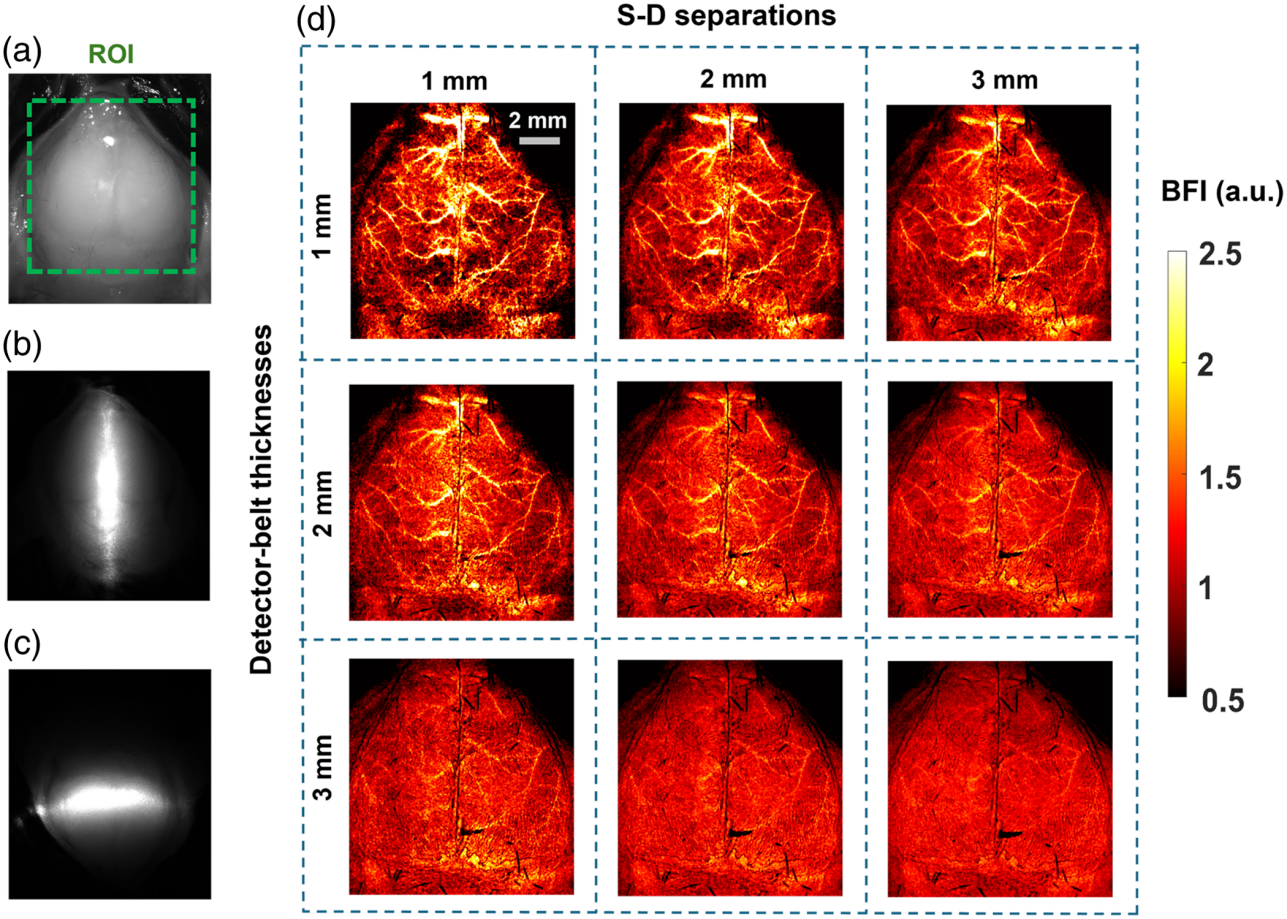
Two-dimensional maps of BFI at different depths in a representative mouse (mouse #7). (a) The selected ROI for BFI mapping on the exposed skull with its scalp retracted. (b) The speckle image with a vertical scanning line on the ROI. (c) The speckle image with a horizontal scanning line on the ROI. (d) 2D maps of BFI with varied S-D separations of 1 to 3 mm and detector-belt thicknesses of 1 to 3 mm.

**Fig. 6:**
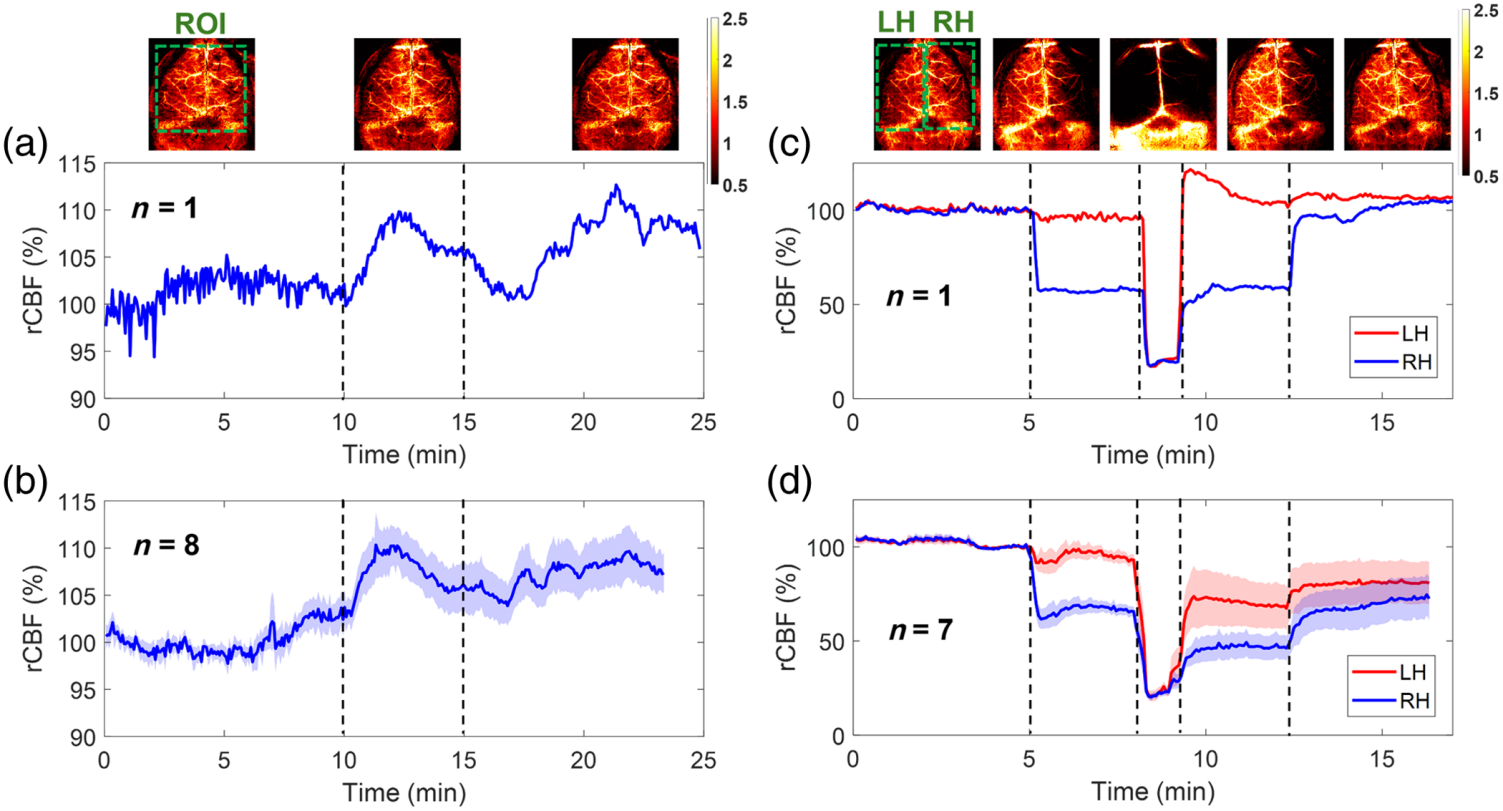
Continuous mapping of rCBF variations during 8% ${\mathrm{CO}}_{2}$ inhalations and transient carotid artery ligations in mice with intact skulls. (a) BFI maps and time-course rCBF changes before, during, and after 8% ${\mathrm{CO}}_{2}$ inhalation in an illustrative mouse (mouse #8). The dash lines separate 10 min of baseline, 5 min of ${\mathrm{CO}}_{2}$ inhalation, and 10 min of recovery, respectively. The ROI for data analysis of rCBF time-course changes is shown in the first BFI map. (b) Group average time-course rCBF changes during 8% ${\mathrm{CO}}_{2}$ inhalations in eight mice (mouse #1 to mouse #8). The error bars represent standard errors. (c) BFI maps and time-course rCBF changes during transient carotid artery ligations in an illustrative mouse (mouse #8). The dash lines separate the 5 min of baseline, 3 min of right carotid artery ligation, 1 min of bilateral ligation, 3 min of left carotid artery release, and 5 min of right carotid artery release. The ROI of LH and RH for data analyses of rCBF time-course changes are shown in the first BFI map. (d) Group average time-course rCBF changes during transient carotid artery ligations in seven mice (mouse #1 to mouse #4 and mouse #6 to mouse #8). The error bars represent standard errors.

**Fig. 7:**
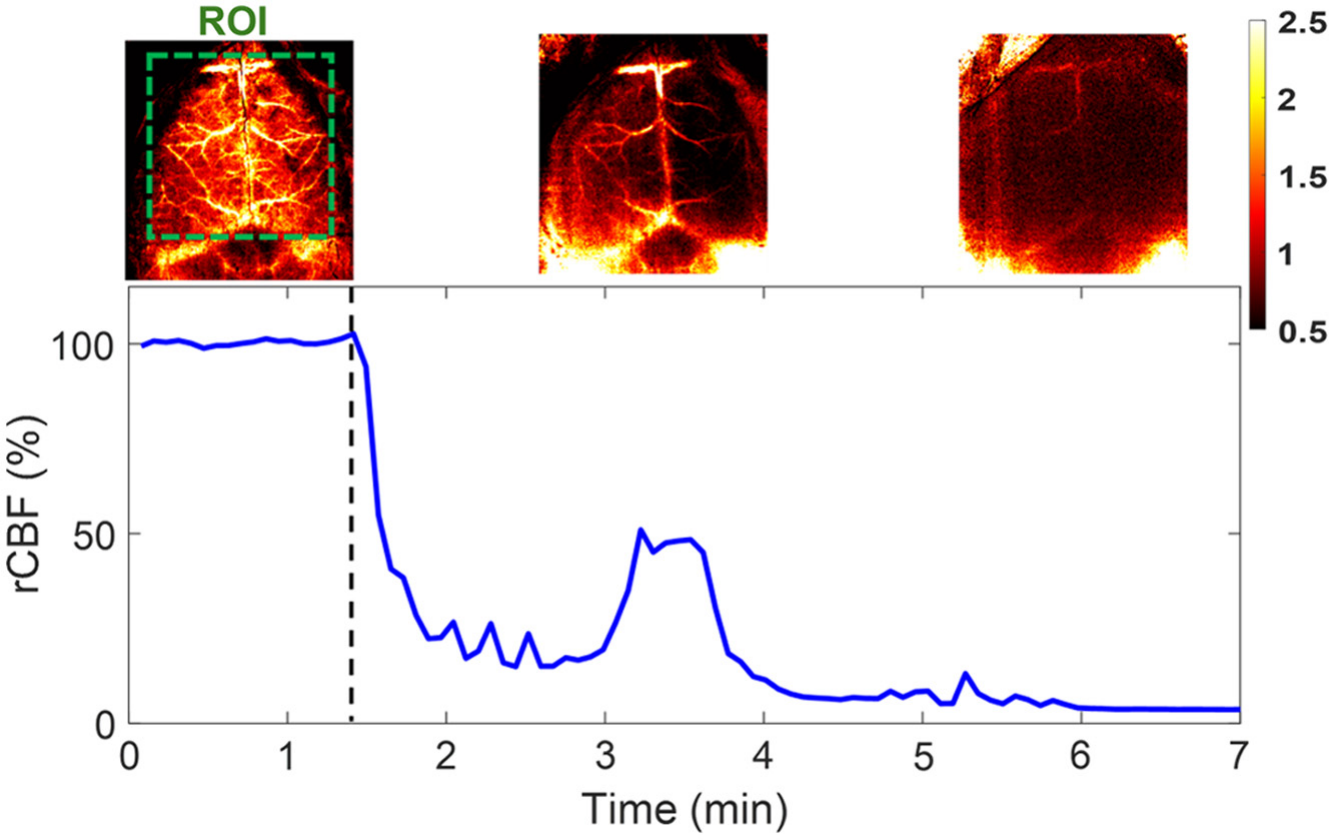
Continuous mapping of rCBF variations during 100% ${\mathrm{CO}}_{2}$ inhalations in mouse #8 with an intact skull. BFI maps and time-course rCBF changes in mouse #8 before and during 100% ${\mathrm{CO}}_{2}$ inhalation until its death. More than a 95% decrease in rCBF was observed at the end of the experiment when mouse #8 died. The dashed line separates the baseline and 100% ${\mathrm{CO}}_{2}$ inhalation phases. The ROI for data analysis of rCBF time-course changes is shown in the first BFI map.

Figure 5 demonstrates the capability of PS-DSCI to generate 2D maps of BFI at different depths in one illustrative mouse (mouse #7). Because the average thickness of adult mouse skulls is about 0.5 to 1 mm^57^ and based on the phantom test results shown in Fig. 4, 2D maps of BFI with varied S-D separations of 1 to 3 mm and detector-belt thicknesses of 1 to 3 mm are reconstructed and shown in Fig. 5(d). The results highlighted that when using smaller S-D separations and thinner detector belts, PS-DSCI generated BFI maps from superficial brain tissues with higher spatial resolutions and greater SNRs. Accordingly, the S-D separation and detector-belt thickness were set to 1 and 2 mm, respectively, for analyzing time-course rCBF changes in Figs. 6 and 7.

#### 3.2.2 PS-DSCI enabled continuous mapping of global rCBF increases in mice with intact skulls during 8% ${\mathrm{CO}}_{2}$ inhalations

Figures 6(a) and 6(b) show time-course changes of rCBF before, during, and after 8% ${\mathrm{CO}}_{2}$ inhalation in one illustrative mouse (mouse #8) and group mice (n=8, mouse #1 to mouse #8,) with intact skulls, respectively. Some animals exhibited noticeable body movements, along with irregular respiration rates and heartbeats, after ${\mathrm{CO}}_{2}$ inhalation, which may contribute to some variations and spikes in regional rCBF. Future experiments will include a pulse oximeter to continuously monitor respiration and heart rates, enhancing our understanding of these physiological and cerebral hemodynamic variations and interactions.

After discontinuing ${\mathrm{CO}}_{2}$ inhalation, rCBF gradually decreased toward baseline; however, it did not fully return to baseline level by the end of our recovery measurement over ∼10  min. Prolonged recoveries following ${\mathrm{CO}}_{2}$ inhalation in rodents have also been reported in other studies with similar experimental protocols.^58^ Nonetheless, longer periods of optical measurements are needed to capture the full recovery of rCBF following ${\mathrm{CO}}_{2}$ inhalation.

Table 3 summarizes group time-course changes in rCBF and corresponding p-values for comparing those changes at different phases of ${\mathrm{CO}}_{2}$ inhalation relative to the baseline (assigning 100%). The average rCBF values at the baseline and recovery phases were quantified using the mean values of data during the entire baseline and recovery phases. The average rCBF values at the 8% ${\mathrm{CO}}_{2}$ inhalation phase were quantified over a 2-min period of the ${\mathrm{CO}}_{2}$ inhalation phase, when peak rCBF increases were observed. The results show that the inhalation of 8% ${\mathrm{CO}}_{2}$ resulted in a significant increase in rCBF (109.98%±2.26%, p=0.012) from the baseline (100%), which lasted to the recovery phase (107.07%±2.36%, p=0.025).

**Table 3:** Group average rCBF changes (mean ± standard error) from their baselines (100%) during 8% ${\mathrm{CO}}_{2}$ inhalations in eight mice (mouse #1 to mouse #8).

Brain region	8% ${\mathrm{CO}}_{2}$ inhalation	Recovery
Global rCBF	109.98% ± 2.26%; p=0.012	107.07% ± 2.36%; p=0.025

#### 3.2.3 PS-DSCI Enabled Continuous Mapping of Regional rCBF Reductions in Mice with Intact Skulls During Transient Carotid Artery Ligations

After recovering from 8% ${\mathrm{CO}}_{2}$ inhalation, eight mice underwent sequential transient carotid arterial ligation surgeries. One mouse (mouse #5) was excluded from the group average results due to its unfortunate death after bilateral ligations. Figures 6(c) and 6(d) show time-course changes of rCBF at the LH and RH before, during, and after sequential transient carotid artery ligations in one illustrative mouse (mouse #8) and group mice (n=7, excluding mouse #5), respectively. Notably, two mice (mouse #2 and mouse #3) out of seven experienced incomplete releases of arterial ligations during the loosening of their knot sutures, leading to incomplete recovery of rCBF. As a result, the average rCBF showed relatively larger variations across subjects during the recovery phases [Fig. 6(d)].

Table 4 summarizes average changes in rCBF, along with the corresponding p-values for comparing these changes during different phases of transient carotid artery ligations to their respective baseline values. The mean rCBF values reported in Table 4 were quantified using the mean values of rCBF data during the entire periods of different phases. As expected, sequential ligations/releases of the right and left carotid arteries led to significant rCBF changes in relevant brain hemispheres, compared with their baselines.

**Table 4:** Group average rCBF changes (mean ± standard error) from their baselines (100%) during sequential transient carotid arterial ligations in seven mice (excluding mouse #5).

Brain region	Right ligation	Bilateral ligation	Release left ligation	Release right ligation
RH rCBF	66.58% ± 3.05%; p=0.018	23.62% ± 1.73%; p=0.018	44.01% ± 5.98%; p=0.018	64.70% ± 10.13%; p=0.018
LH rCBF	93.25% ± 2.89%; p=0.091	26.07% ± 1.49%; p=0.018	67.15% ± 1.25%; p=0.043	77.52% ± 11.16%; p=0.063

#### 3.2.4 PS-DSCI enabled continuous mapping of sharp rCBF decrease in a mouse with an intact skull during 100% ${\mathrm{CO}}_{2}$ inhalation

Following the artery ligations/releases, one mouse (mouse #8) was subjected to 100% ${\mathrm{CO}}_{2}$ inhalation to test the capability of PS-DSCI for capturing rapid rCBF decreases until the animal’s death. Figure 7 shows time-course changes of rCBF before (assigned 100%) and during 100% ${\mathrm{CO}}_{2}$ inhalation over ∼5.5  min in mouse #8. A significant reduction in rCBF was observed immediately after inhaling 100% ${\mathrm{CO}}_{2}$, followed by a transient increase at 3 to 4 min when the animal had long, deep, gasping breaths. Subsequently, rCBF steadily declined to ∼5% of its baseline (100%) by the time when the animal was declared dead.

#### 3.2.5 PS-DSCI enabled noninvasive and continuous monitoring of rCBF variations in a mouse with an intact head

**Fig. 8:**
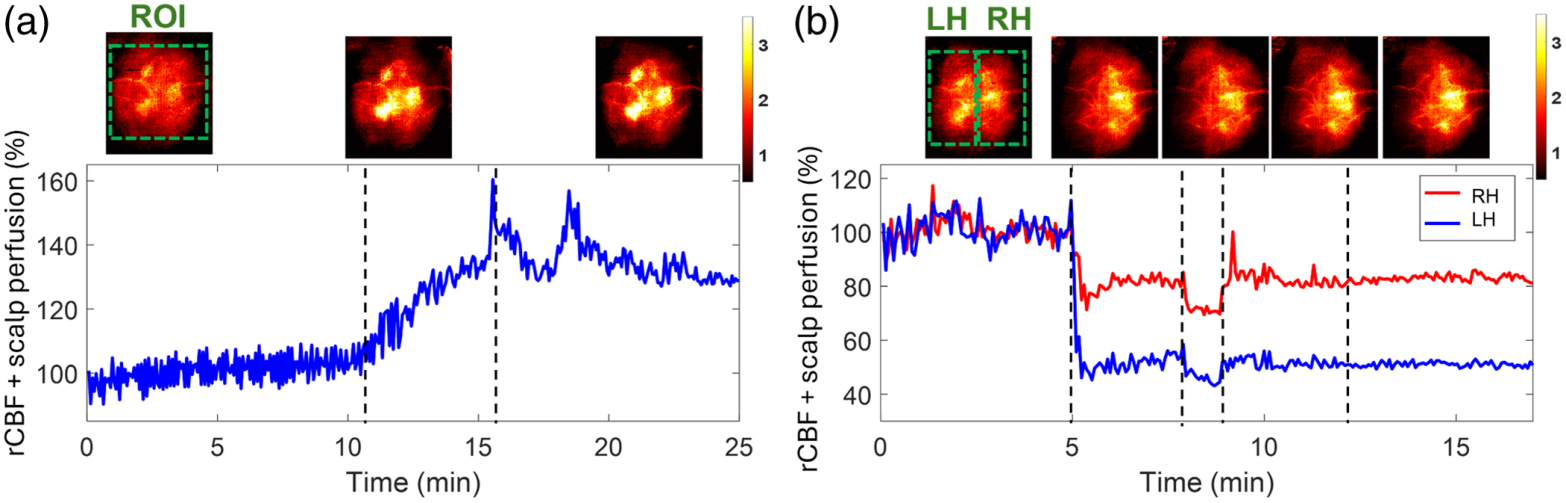
Continuous mapping of rCBF variations during 8% ${\mathrm{CO}}_{2}$ inhalations and transient carotid artery ligations in mouse #9 with an intact head. (a) BFI maps and time-course rCBF changes before, during, and after 8% ${\mathrm{CO}}_{2}$ inhalation in mice with intact heads. The dash lines separate 10 min of baseline, 5 min of ${\mathrm{CO}}_{2}$ inhalation, and 10 min of recovery, respectively. The ROI for data analysis of rCBF time-course changes is shown in the first BFI map. (b) BFI maps and time-course rCBF changes during transient carotid artery ligations in a mouse with an intact head. The dashed lines separate the 5 min of the baseline, 3 min of the left carotid artery ligation, 1 min of the bilateral ligation, 3 min of the right carotid artery release, and 5 min of the left carotid artery release. The ROI of LH and RH for data analysis of rCBF time-course changes are shown in the first BFI map.

One mouse (mouse #9) with an intact head (without scalp retraction) underwent 8% ${\mathrm{CO}}_{2}$ inhalation and transient carotid artery ligations to evaluate the capability of PS-DSCI for noninvasive and continuous monitoring of rCBF variations. Given the increased total top layer thickness of the scalp and skull, the S-D separation of 2 mm and detector-belt thickness of 2 mm were used for the reconstruction of BFI maps. Figure 8(a) shows the results from the continuous mapping of rCBF variations during 8% ${\mathrm{CO}}_{2}$ inhalation in mouse #9. rCBF increased to 132.0%±2.7% and 135.2%±6.4% (mean ± standard deviation) at the maximum increase of 8% ${\mathrm{CO}}_{2}$ inhalation phase and during the recovery phase from its baseline (100%), respectively. These rCBF changes during 8% ${\mathrm{CO}}_{2}$ inhalation [Fig. 8(a)] were larger than those from the mice without scalp [Figs. 6(a) and 6(b)], likely due to the overlayed blood flow elevations from both the scalp and cortex in response to ${\mathrm{CO}}_{2}$ in mouse #9.

The transient carotid artery ligations induced similar rCBF reductions in mouse #9 [Fig. 8(b)], compared with those from the mice without scalp [Figs. 6(c) and 6(d)]. We noted again that the carotid artery ligations were not released completely in mouse #9, leading to incomplete rCBF recoveries in both hemispheres. Nevertheless, rCBF variations during artery ligations are clearly discernible. In addition, the partial volume effect of the scalp rendered cerebral vasculatures in the reconstructed BFI maps.

## 4 Discussion and Conclusions

The vitality and functionality of the brain tissue are highly dependent on proper CBF circulation. Continuous imaging of CBF holds promise for the diagnosis and therapeutic management of many cerebral and neurovascular diseases. To meet such a critical need, we developed a DMD-based, line scanning, depth-sensitive PS-DSCI technique for noncontact and continuous imaging of CBF with remarkably improved spatiotemporal resolution (Figs. 1–3). Compared with point scanning in scDCT, line scanning in PS-DSCI significantly increases the spatiotemporal resolution of CBF imaging while reducing computation time and storage requirements for image reconstructions (Table 1). Moreover, the DMD enables the creation of diverse and rapid scanning patterns, including vertical and horizontal line scanning, parallel line scanning, and multi-point scanning, to achieve an optimal spatiotemporal resolution.

DMD-based illumination and imaging methods have become state-of-the-art in biomedical optics, offering programmability, compactness, speed, and precision that are invaluable for advanced imaging applications. DMD has been used in a variety of optical imaging modalities, including spatial frequency domain imaging,^59^^,^^60^ optical diffraction tomography,^61^^–^^63^ fluorescence microscopy,^64^ diffuse optical tomography,^65^^,^^66^ and hyperspectral imaging.^67^ Interested readers are encouraged to explore these publications to investigate the benefits and limitations of DMD for different applications. Among these applications, this study introduces a new use of DMD: generating fast line-shaped scanning in PS-DSCI, which significantly enhances spatiotemporal resolution compared with the previous point scanning method used in scDCT.

In designing the PS-DSCI prototype (Fig. 1), the DMD was illuminated with semi-collimated light to create a scanning pattern with a large depth of focus. This design allows for a flexible working distance while preventing overlaps of diffraction patterns.^68^^,^^69^ Although the use of collimated light limits the scanning ROI, the PS-DSCI prototype enables CBF imaging in head-simulating phantoms and small rodents (Figs. 4–8). A diverging lens may be added to the projection path to enlarge the scanning ROI for future applications in larger subjects.

In analyzing collected PS-DSCI data, deformations of line-shaped sources were observed, resulting from the Gaussian distribution of light and curvature of the target tissue surface. We innovatively developed new algorithms to extract the source properties at each scanning position. Based on the extracted source properties, elliptical shaped detector areas/belts with predefined S-D separations were used to address deformations of line-shaped scanning, thus minimizing BFI reconstruction artifacts (Fig. 3). The S-D separations and detector-belt thicknesses can be modified for reconstructing BFI images at different depths and with different SNRs.

To evaluate the depth sensitivity of PS-DSCI in mapping flow distributions at different depths, head-simulating phantoms with known optical and geometrical properties were fabricated and scanned (Fig. 4). Results demonstrate that with the optimized S-D separations and detector-belt thicknesses, our PS-DSCI enables resolving the infinity-shaped channel of intralipid particle flow contrasts at the depths up to ∼3  mm in the two head-simulating phantoms with top layer thicknesses of 1 and 2 mm. These phantom experiments provide guidance to optimize the PS-DSCI for obtaining the best image quality in rodents with known head geometries (Figs. 5–8).

The capabilities of the PS-DSCI for 2D mapping of BFI at different depths and continuous monitoring of global and regional rCBF alterations were examined in nine adult mice with intact skulls (mouse #1 to mouse #8) or intact heads (mouse #9) during a variety of pathophysiological manipulations (Table 2). Based on the variation in top layer thicknesses of the scalp/skull, the S-D separations and detector-belt thicknesses were optimized for the reconstruction of BFI maps at different depths (Figs. 5–8).

The results in mice with intact skulls but retracted scalps (mouse #1 to mouse #8) show that the inhalation of 8% ${\mathrm{CO}}_{2}$ resulted in a significant global rCBF increase [109.98%±2.26%, Fig. 6(b) and Table 3] and sequential ligations/releases of the right and left carotid arteries led to significant regional rCBF variations in relevant brain hemispheres [Fig. 6(d) and Table 4]. These results meet pathophysiological expectations and are generally consistent with previous studies in adult rodents using scDCT, LSCI, MRI, and PET and similar experimental protocols.^42^^,^^55^^,^^70^^–^^74^ At the end of the experiment, mouse #8 was subjected to 100% ${\mathrm{CO}}_{2}$ inhalation, during which a remarkable reduction in rCBF was observed immediately (Fig. 7). Upon the animal’s death, rCBF dropped to 5% of its baseline level (100%), demonstrating the sensitivity of PS-DSCI in detecting a minimal CBF level.

One mouse (mouse #9) with an intact head underwent 8% ${\mathrm{CO}}_{2}$ inhalation and transient carotid artery ligations to evaluate the capability of PS-DSCI for noninvasive monitoring of rCBF variations. Results show larger rCBF changes during 8% ${\mathrm{CO}}_{2}$ inhalation [Fig. 8(a)], compared with those from the mice without scalp [Fig. 6(b)], which is likely due to the overlayed blood flow elevations from both the scalp and cortex in response to ${\mathrm{CO}}_{2}$ in mouse #9 with an intact head. The transient carotid artery ligations induced similar regional rCBF reductions in mouse #9 [Fig. 8(b)], compared with those from the mice without scalp [Figs. 6(c) and 6(d)]. Even though the partial volume effect of scalp rendered cerebral vasculatures, rCBF variations during carotid artery ligations are clearly discernible.

Some limitations in the present study have been identified, which will be addressed in future research. The current PS-DSCI prototype utilizes a camera with a frame rate of 24 fps (C11440-42U40, Hamamatsu), resulting in a sampling rate of 0.24 Hz when scanning a total of 100 lines. Although this sampling rate is 25 times faster than point-source scanning, it can be further improved by optimizing both the illumination and detection sides. On the detection side, using advanced cameras with faster frame rates can improve the sampling rate, though it also increases the cost. On the scanning side, reducing the number of scanning lines enhances temporal resolution but comes at the cost of reduced spatial resolution. In addition, because the programmable DMD enables the creation of diverse scanning patterns, exploring alternative patterns can further increase the sampling rate. Moreover, compressive sensing can enhance the sampling rate by requiring fewer scanning lines and incorporating sparsity-based algorithms.^75^^–^^77^ These improvements in sampling rates are expected to enable PS-DSCI for the rapid, depth-sensitive mapping of brain FC, which will be the focus of our future research.

In the study of global cerebral responses to ${\mathrm{CO}}_{2}$ inhalation, rCBF did not fully recover to baseline levels by the end of the recovery measurements, which lasted ∼10  min [Figs. 6(a) and 6(b)]. Studies using different modalities such as photoacoustic microscopy, functional MRI, and pseudo-continuous arterial spin labeling MRI have shown that cerebral hemodynamic responses to ${\mathrm{CO}}_{2}$ inhalation are dynamic and ${\mathrm{CO}}_{2}$-level dependent.^58^^,^^78^^,^^79^ The removal of ${\mathrm{CO}}_{2}$ from the blood after discontinuing ${\mathrm{CO}}_{2}$ inhalation is significantly slower than its dissolution into the blood via the lung alveoli. In addition, the gradual accumulation of anesthetics contributes to a progressive increase in blood flow, which is most pronounced during the recovery phase. Prolonged recoveries following ${\mathrm{CO}}_{2}$ inhalation in rodents have also been observed in other studies.^58^^,^^79^ Therefore, a longer period of optical measurement is necessary to fully capture the recovery of rCBF after the cessation of ${\mathrm{CO}}_{2}$ inhalation.

In the study of regional cerebral responses to transient carotid arterial ligations, two out of seven mice experienced incomplete release of arterial ligations during the loosening of their knot sutures, leading to incomplete recovery of rCBF [Figs. 6(c) and 6(d)]. This procedure has proven more reliable in rats, which have larger arteries, making ligation and recovery more consistent.^42^ Due to the smaller size of the arteries in mice, the knot and the pressure applied during ligation placed greater stress on the arteries, making them more likely to remain ligated. Nevertheless, PS-DSCI enabled the detection of rCBF variations during both the tightening and loosening of the knot suture, helping to confirm the ligation and release of the artery. This information will contribute to the improvement of ligation techniques.

In summary, to meet the need for fast, high-density, and continuous imaging of CBF at different depths, we have developed a low-cost, DMD-based, line scanning, depth-sensitive PS-DSCI technique. Compared with the point scanning in conventional scDCT, the line scanning in the new PS-DSCI significantly increases the spatiotemporal resolution of CBF imaging and reduces the computation time and storage for image reconstructions. New algorithms have been developed to address deformations of line-shaped scanning, thus minimizing BFI reconstruction artifacts. The capabilities of the PS-DSCI for 2D mapping of BFI at different depths and continuous monitoring of global and regional rCBF alterations were examined in head-simulating phantoms and adult mice with known optical and geometrical properties of heads. The results from PS-DSCI are consistent with previous studies in adult rodents using other technologies and similar experimental protocols. Importantly, the high spatiotemporal resolution of PS-DSCI holds the promise for depth-sensitive mapping of brain functional activity.

Future studies will aim to develop and optimize a low-cost, fast, mobile, and user-friendly PS-DSCI system with improved features, including higher sampling rates, larger ROIs, greater penetration depth, enhanced SNR, and reduced computation time. This will enable real-time cerebral and functional imaging in larger animals (e.g., rats and piglets) and human infants. Higher sampling rates can be achieved through the previously mentioned methods. Optimizing the projection optics in front of the DMD will allow for a larger scanning ROI. Greater penetration depth and enhanced SNR can be attained by utilizing longer-wavelength light (e.g., 1064 nm) with higher illumination power, alongside an InGaAs camera for better detection of longer wavelengths.^80^ In addition, computation time for data analysis can be reduced by implementing more efficient parallel algorithms. Given our successful development of scDCT for cerebral imaging in mice, rats, neonatal piglets, and human neonates^29^^–^^36^^,^^81^ and the technological similarities, we do not anticipate significant challenges in scaling up the PS-DSCI. In addition, we may explore the use of boundary data collected by PS-DSCI in conjunction with perturbation models based on Monte Carlo simulations to reconstruct 3D maps of rCBF,^47^ which could help reduce partial volume effects from the scalp and skull. Moreover, our study lays the groundwork for future investigations involving a larger number of subjects to explore biological variability and investigate the pathophysiological and interventional mechanisms of various cerebral diseases.

## Data Availability

All codes and data supporting the findings of this study are available from the corresponding author upon reasonable request.
